# Regional patterns of postglacial changes in the Palearctic mammalian diversity indicate retreat to Siberian steppes rather than extinction

**DOI:** 10.1038/srep12682

**Published:** 2015-08-06

**Authors:** Věra Pavelková Řičánková, Jan Robovský, Jan Riegert, Jan Zrzavý

**Affiliations:** 1 Department of Zoology, Faculty of Science, University of South Bohemia, České Budějovice, Czech Republic

## Abstract

We examined the presence of possible Recent refugia of Pleistocene mammalian faunas in Eurasia by analysing regional differences in the mammalian species composition, occurrence and extinction rates between Recent and Last Glacial faunas. Our analyses revealed that most of the widespread Last Glacial species have survived in the central Palearctic continental regions, most prominently in Altai–Sayan (followed by Kazakhstan and East European Plain). The Recent Altai–Sayan and Kazakhstan regions show species compositions very similar to their Pleistocene counterparts. The Palearctic regions have lost 12% of their mammalian species during the last 109,000 years. The major patterns of the postglacial changes in Palearctic mammalian diversity were not extinctions but rather radical shifts of species distribution ranges. Most of the Pleistocene mammalian fauna retreated eastwards, to the central Eurasian steppes, instead of northwards to the Arctic regions, considered Holocene refugia of Pleistocene megafauna. The central Eurasian Altai and Sayan mountains could thus be considered a present-day refugium of the Last Glacial biota, including mammals.

Last Glacial landscape supported a unique mix of large species, now extinct or living in non-overlapping biomes, including rhino, bison, lion, reindeer, horse, muskox and mammoth^1^. The so called “mammoth steppe”^2^,^3^,^4^ community thrived for approximately 100,000 years without major changes, and then became extinct by the end of Pleistocene, around 12,000 years BP^5^,^6^.

Diversity of climatic conditions and vegetation belts in Eurasia resulted in great regional differences in timing and degree of mammoth-steppe fauna regional extirpation or global extinction (we use the term extirpation for local extinctions and term extinction for global extinctions afterwards)^7^,^8^,^9^,^10^,^11^,^12^. As the climate became milder by the end of the Last Glacial, human population densities increased and Pleistocene megafauna survived in areas unaffected by vegetation changes and/or human hunting pressure^10^. The most widely held concept is that the cold-adapted Pleistocene megafauna retreated to the treeless North inhabited only by a sparse human population. This “retreat to the North” scenario^13^ is supported by a significant delay of megafauna extinction in North–East Asia: many iconic species of large herbivores (e.g. mammoth *Mammuthus primigenius*, horse *Equus* spp., and bison *Bison priscus*) existed in northern Yakutia for a long time during the Holocene, so this region is often considered a Holocene refugium of the mammoth-steppe biome^8^,^10^.

At present, there are remaining patches of steppe in the Far North scattered in boreal forest and tundra habitats^14^ but this ecosystem supports only few mammalian species. Moreover, the present-day mammalian fauna in Yakutia lacks the typical steppe elements such as horse (*Equus* spp.), saiga antelope (*Saiga tatarica*) or steppe pika (*Ochotona pusilla*)^15^. On the contrary, the glacial-like ecological structure of mammalian communities remains preserved in the present-day ecosystems of the Altai–Sayan region in central Eurasia^16^. The Recent distribution of steppe species seems to correspond to the continental/oceanic longitudinal gradient of decreasing precipitation and pronounced seasonal variation toward the center of a continent. The longitudinal and latitudinal gradients then interplay in determining the precise distribution of a Last Glacial species, depending on the species’ ecological requirements^17^,^18^.

The excellent mammalian fossil record offers the opportunity to examine regional-specific extinction patterns of both large and small mammals^19^ as the large mammals alone may not be the best marker of past environmental changes^20^,^21^. By comparing extinction rates and Recent ranges of widespread Last Glacial species in various regions of the Palearctic we aim to detect the presence of possible Recent refugia of the Last Glacial mammalian faunas.

## Results

### Similarity of Recent and Last Glacial faunas

**Figure 1:**
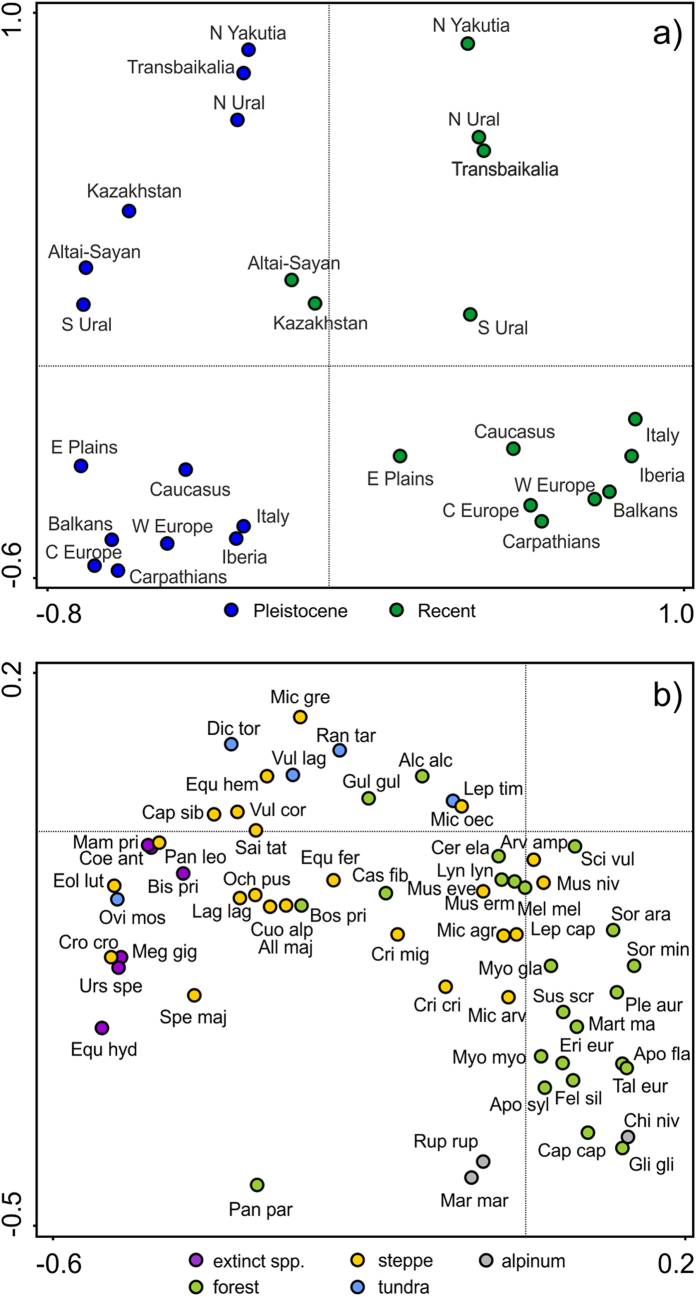
PCA scores, according to the presence/absence of the 64 widespread Last Glacial species in each region. We show diagrams for **a**) regions and **b**) species. The first two axes (first axis = horizontal axis, second axis = vertical axis) describe 52% of variance. In (**a**) Last Glacial and Recent faunas of each region are treated separately and regions thus appear twice. For species abbreviations see Supplementary Dataset S1. *Vulpes vulpes*, *Canis lupus* and *Ursus arctos* were invariably present in all examined regions.

The PCA analysis revealed that Last Glacial and Recent faunas form two groups separated along the first (horizontal) axis (Fig. 1a). The Recent Altai–Sayan and Kazakhstan regions, positioned in the middle of the Last Glacial–Recent gradient, show species compositions more similar to their Pleistocene counterparts. The gradient between Last Glacial and Recent faunas (Fig. 1b) was determined by the presence/absence of globally extinct species (e.g. woolly mammoth *Mammuthus primigenius*, steppe bison *Bison priscus*), steppe species (e.g. wild horse *Equus ferus*, Siberian ibex *Capra sibirica*, steppe pika *Ochotona pusilla*), and forest species (e.g. Eurasian pygmy shrew *Sorex minutus*, yellow-necked field mouse *Apodemus flavicollis*, red squirrel *Sciurus vulgaris*). The second (vertical) axis in Fig. 1b reflects the difference between Arctic and temperate faunas: the Recent Arctic faunas were characterized by the presence of tundra species (e.g. Arctic fox *Vulpes lagopus*, Arctic lemming *Dicrostonyx torquatus*), and the Recent European faunas by temperate forest species (e.g. western roe deer *Capreolus capreolus*, wild boar *Sus scrofa*). Both the Last Glacial and Recent faunas form three groups: (i) southern faunas including Mediterranean peninsulas, Caucasus, East European Plain, Carpathians, West and Central Europe; (ii) steppe group including Altai–Sayan, Kazakhstan and South Ural; (iii) eastern–Arctic group including North Yakutia, North Ural and Transbaikalia (Fig. 1a). Similar results were obtained by Principal Coordinates Analysis (where the Pleistocene and Recent faunas showed gradient on both axes) and cluster analysis (see Supplementary Fig. S1,S2). According to results of Analysis of Similarity (ANOSIM), Recent Altai–Sayan and Kazakhstan faunas were most similar to the group of Pleistocene Altai–Sayan, Kazakhstan, North Ural, South Ural, North Yakutia and Transbaikalia faunas (Supplementary Table S2). Moreover, Recent Altai–Sayan and Kazakhstan were significantly different from the group of both Pleistocene and Recent Mediterranean peninsulas, Caucasus, East European Plain, Carpathians, West and Central Europe faunas (Supplementary Table S2).

### Comparison of species extinctions between Last Glacial and Recent faunas

Of the 364 mammalian species found in the fossil record of the 14 examined regions, 44 species (12%) went extinct in the whole Palearctic during the Last Glacial and Holocene. Nineteen of the 44 extinctions (43%) occurred during the Last Glacial/Holocene transition.

**Figure 2:**
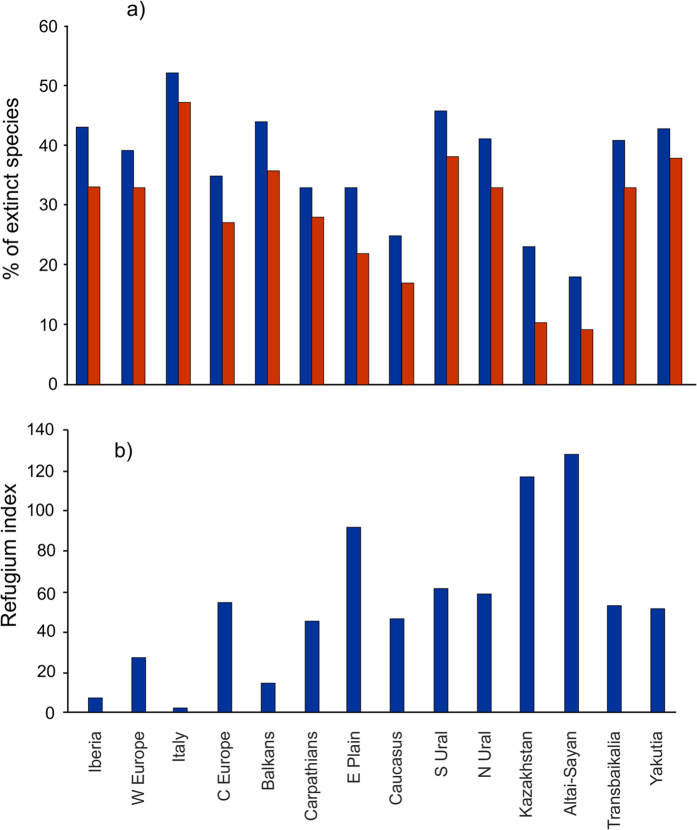
(a) Proportions (%) of extirpated species for each region. All species (including globally extinct species) are marked by blue columns, extant species by red ones. **(b) Refugium index for each of the examined regions.**

Comparison of Last Glacial and Recent complete mammalian faunas (including globally extinct species) revealed that the lowest number of extirpated species were in the Altai–Sayan region (18% of Last Glacial species), followed by Kazakhstan (23%) and Caucasus (25%). The East European Plain and Carpathian Mountains lost 33% of the Last Glacial species each, Central Europe 35%, and West Europe 39% (Fig. 2a). The highest proportion of extirpated species was in the Yakutia (43%), South Ural (46%) and Italy (52%) regions (Supplementary Table S2).

Comparison of Last Glacial and Recent mammalian faunas excluding globally extinct species revealed a similar pattern (Fig. 2a). The smallest number of extirpated species was found in Altai–Sayan (9% of Last Glacial species), followed by Kazakhstan (10%), Caucasus (17%), and East European Plain (22%). Central Europe lost 27% of Last Glacial species and the Carpathian Mountains 28%. The highest proportion of extirpated species was observed in Yakutia (38%), South Ural (38%) and Italy (47%) (Supplementary Table S2).

### Refugium index

Comparison of the “refugium index” (Ri) values among the examined regions revealed the highest value for the Altai–Sayan (Ri = 128), Kazakhstan (Ri = 117), and East European Plain (Ri = 92) regions (the maximum possible refugium index value is 153). The lowest value of the index (Ri = 3) was found in the Italian region (Fig. 2b). The high level of survival of the Last Glacial species in the three Central Eurasian regions (with maximum in Altai–Sayan) is caused by regional survival of otherwise extirpated Last Glacial species there (e.g. Mongolian saiga *Saiga borealis*, reindeer *Rangifer tarandus*, wild horse *Equus ferus*, dhole *Cuon alpinus*, steppe pika *Ochotona pusilla*, narrow-headed vole *Microtus gregalis*). Arctic regions display low species numbers and therefore low refugium index values, but the relative index value per species present in the region in question might be rather high: e.g. Yakutia displays a relatively low refugium index (Ri = 52) but the relative refugium index reaches the same value (ri = 3) as the Altai–Sayan region.

### Regional patterns in Recent and Last Glacial mammal distributions

**Figure 3:**
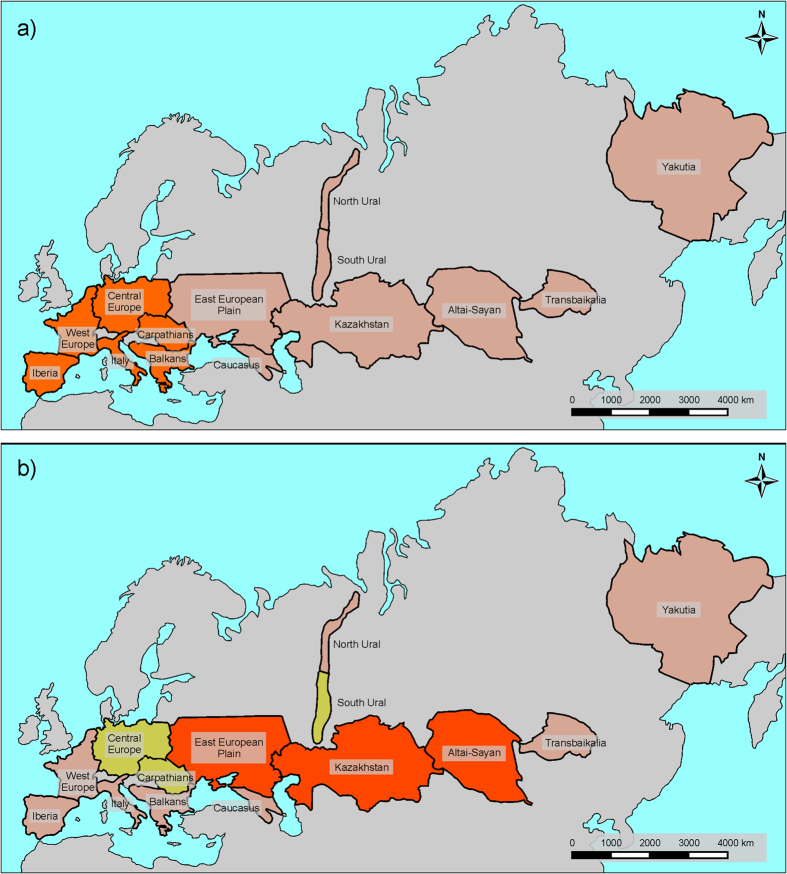
Regions sharing high numbers of extirpated (a) surviving (b) Last Glacial species. Regions with the highest numbers are marked orange, regions with lower numbers yellow, other regions light brown. The map was created using CorelDRAW(R) Home and Student X5 v.15.1.0.588, (c) 2010 Corel Corporation.

Based on analysis of the 52 “core species” (species once distributed in at least four regions examined, displaying clear continuity of Pleistocene and Recent distribution in at least one region, and now surviving in at least one region), there are obvious similarities in regional extinction/survival patterns. The Palearctic faunas seem to form two higher-level regional groupings: (i) all European regions (Central Europe, West Europe, Carpathians, Iberia, Italy, Balkans) with 10–16 shared *extirpated* species (Fig. 3a, Table 1), versus (ii) Altai–Sayan–Kazakhstan–East European Plain “super-region” with highest shared numbers (19–21) of *surviving* Last Glacial species (Fig. 3b, Table 2), followed by the Carpathians, Central Europe and South Ural regions with 12–18 shared surviving species. This indicates there is a general west–east difference in the regional extinction rates (Table 1).

### Range retraction patterns: the fate of extirpated species

**Figure 4:**
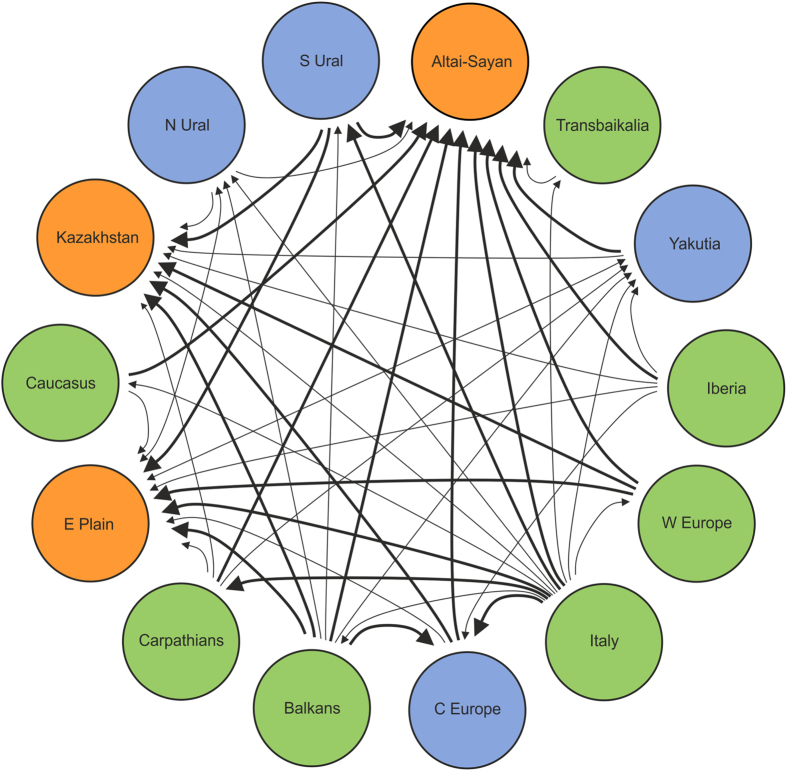
Directions of the Holocene retractions of Last Glacial species (note that “X → Y” indicates retraction from X to Y, not migration from X to Y). Thin lines mean 7–12 species retractions, thick lines more than 13 species retractions. The most important refugia for Pleistocene species are marked in orange, the less important are marked in blue, and other regions are green.

More detailed analysis of Last Glacial/Recent areal changes suggests that many species extirpated in Europe, Caucasus, and South Ural are still surviving in the Altai–Sayan–East European Plain–Kazakhstan “super-region”, most prominently in Altai–Sayan. The northernmost regions (Yakutia, North Ural), together with Central Europe and South Ural, represent less important refugia for the extirpated Last Glacial mammals (Fig. 4, Table 3).

## Discussion

The local extinction patterns of Last Glacial mammalian faunas in the Palearctic Realm vary greatly. There is, however, an obvious general trend in regional-specific extinction rates: the Last Glacial species extirpated especially in the western Palearctics (most prominently in southern Europe) but have often survived in the central Palearctic continental regions, most prominently in Altai–Sayan (followed by Kazakhstan and East European Plain). The Altai–Sayan region retained most of its Pleistocene mammalian fauna; only one of the Recent megafauna species (the muskox) went extinct there (as well as in all Palearctic regions). The “refugium index” value of this central Eurasian region is almost twice as high as those of the other regions. These central Eurasian mountain ranges thus could be considered a present-day refugium of Last Glacial mammals. Nevertheless, continuity of glacial species occurrence in central Eurasia during Holocene could not be reliably estimated from the fossil record. More phylogeographic studies of formerly widespread Last Glacial species would help to elucidate the biogeographic history of presumed refugia.

The originally wide Last Glacial species distribution ranges were usually retracted eastwards, into the center of the continent, instead of northwards. Longitudinal gradient of oceanicity–continentality played an important role in the evolution of glacial ecosystems; it was clearly more important than the latitudinal one in determining range retractions and extirpations of the mammoth-steppe fauna. A substantial part of the mammoth-steppe mammalian fauna originated on the steppes of inner Eurasia^22^,^23^ some other species have been proposed to originate in Beringia^24^. It has been suggested that the boreal forest belt that originally separated the central Eurasian steppe from the Arctic tundra vanished as the climate became drier around 460,000 years BP, and a new type of biome, the “tundra-steppe” or “mammoth steppe”, evolved. The increased aridity, cooling and continentality then allowed species of Arctic tundra origin to disperse south- and southwestwards, whereas the species of steppe origin spread into northern and western regions of the Palaearctic^23^, together forming the “mammoth-steppe” mammalian fauna.

**Figure 5:**
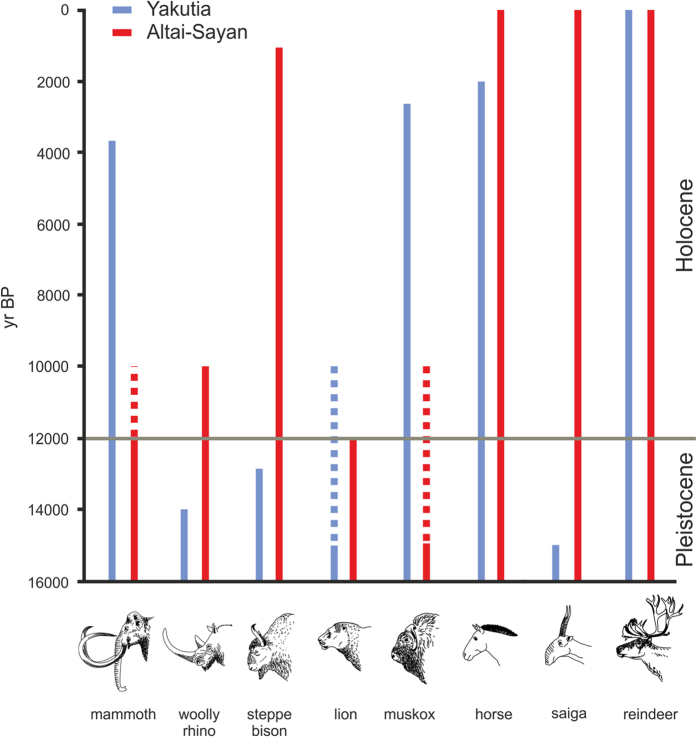
Comparison of the latest extinction dates of megafauna species in Yakutia and Altai–Sayan. 8,50,51,52,53Reproduced with permission of the copyright owner Petr Hrabina.

Although North Asia (incl. North-Central Siberia) has been considered a Holocene refugium of the Last Glacial megafauna^10^,^25^, the Arctic regions display, quite surprisingly, relatively high extinction rates (Yakutia lost 43% of all mammalian species). Some “iconic” species of large herbivores (e.g. mammoths, horses or bisons) survived there for a long time during the Holocene (Fig. 5). Despite significant delay in the megafauna extinctions, the Recent mammalian fauna of Northern Yakutia differs greatly from the Pleistocene assemblages^16^, North-East Asia probably lost its refugial character during further Holocene climate changes^26^,^27^,^28^.

Numerous recent paleoecological studies suggest that better climatic analogues of the Last Glacial period can be found in southern Siberia^29^,^30^,^31^. Vegetation studies have shown that the Altai–Sayan region represents the closest modern analogy to the Last Glacial environments. A close similarity between glacial pollen samples from Central Europe and modern surface-pollen spectra from the Altai–Sayan region has been demonstrated^32^,^33^. Fossil pollen spectra from the Altai and adjacent regions indicate little difference between modern biomes in this region and those reconstructed for the Last Glacial Maximum^34^. The Altai–Sayan mountains are currently inhabited by mollusc assemblages that were characteristic of full-glacial environments across large areas in Eurasia but went extinct in the regions that experienced considerable climatic change, namely in Europe^35^. The ecological structure of the present-day Altai–Sayan mammalian communities closely resembles the Pleistocene assemblages of northern Eurasia^16^. Detailed analysis of the Altai Late Pleistocene assemblages of small mammals revealed that no significant changes occurred between the cold phase of the Pleistocene and the Holocene^36^.

Our results support the “retreat to the central steppes” scenario proposed by climatic and vegetation studies^26^,^28^,^37^. Afforestation of much of western and central Europe began already in the late Last Glacial^11^, whereas farther east it did not occur until the Holocene because of the much drier climate. The Altai–Sayan region is too far inland to have been affected by the enhanced monsoonal rainfall, and its south-eastern parts are still covered by grassland. Mammoth-steppe fauna has thus been preserved in the Altai–Sayan Mountains where humans were not abundant and environmental changes since the Pleistocene were very limited^28^,^38^.

However, the extinction data of the well-known megafauna species, such as mammoth or woolly rhinoceros, did not show significant delay in the Altai–Sayan region in comparison to other Palearctic regions^8^,^39^,^40^. However, the smaller megafauna species survived in Altai–Sayan well into the Holocene (i.e. giant deer or steppe bison) or did not go extinct at all (i.e. wild horse, saiga antelope, reindeer; Fig. 5). The apparent lack of delay in giant herbivore extinctions could be obscured by the fact that Last Glacial and early-Holocene deposits of the refugial south-eastern, steppe parts of the Altai–Sayan region remain poorly investigated^16^,^40^. Indeed, climate models predicted suitable conditions for mammoth survival in eastern Altai–Sayan up to mid-Holocene^41^.

The Pleistocene-Holocene transition was marked by substantial decline of local species richness and radical change in composition of local communities^16^,^21^. The major causes of the postglacial changes in Palearctic mammalian diversity were not extinctions but rather radical shifts of species distribution ranges, a pattern obscured by considering only the global extinction of a few “iconic” megafauna species. The examined Palearctic regions lost 12% of their mammalian species during the last 109,000 years, while megafaunal extinction in the Palearctic has been estimated at 37%^42^. Another continent inhabited by humans during Late Pleistocene, Africa, lost 25% of its megafauna^43^ whereas continents colonized by humans at the end of Pleistocene suffered more megafaunal extinction (i.e. 69% in North America and South America lost 80% of its megafaunal genera)^42^.

Almost half of global extinctions in the Palearctic Realm occurred during a relatively short period of the Pleistocene–Holocene transition whereas nearly all of the documented North American extinctions occurred around Pleistocene-Holocene transition^44^. As in many analyses of Late Quaternary extinctions, interpretation is impeded by lack of data on biotic responses to previous deglaciations. In any case, the Altai–Sayan region offered a suitable refuge for the steppe species, and thanks to its high elevation and topographic heterogeneity also for some tundra species, forming unique ecosystems that preserve important portions of the Pleistocene biota.

## Methods

### Regional faunas

To compare species composition of Recent and Last Glacial faunas, lists of mammalian species for 14 regions have been collected (Supplementary Table S3). The areas were selected to cover most of the Palearctic Realm above 35° N (excl. regions close to the Sino-Japanese Realm^45^ and to include the well documented fossil-mammalian Last Glacial localities.

### Time Periods

The “Recent” mammalian faunas refer to the interval from now to approximately the 16^th^ century AD in order to respect the IUCN definition of “recent extinction”^46^. We included very recently extinct or extirpated species in the dataset. The Last Glacial refers here to the last glacial period of the Late Pleistocene, corresponding with the Weichselian Glaciation. The Last Glacial faunas were then dated from approximately 109,000 to 12,000 years BP, i.e. they included the time interval from MIS 5d to MIS 2^47^,^48^ which is well defined in the geological/fossil record^19^,^49^. As we focused on the processes of the Pleistocene/Holocene transition in local extinction analyses, the dataset for the whole Last Glacial period was limited to species documented for the period of final margin of the Last Glacial i.e. for around 12,000 years BP in at least one of the examined regions.

### Species

Presence/absence of 372 mammalian species in Recent and Last Glacial time periods of each region were recorded (for details about species list, taxonomy, and distribution see^15^ and Supplementary Datasets S1,S3, two exceptions were the inclusion of †*Sorex kennardi* in *S. tundrensis* s. l. and fusing of *Ovis gmelini* with *O. vignei* into *O. gmelini* s. l.).

### Similarity of Recent and Last Glacial faunas

To visualize the overall similarity of Recent and Last Glacial faunas, we used all 14 regions as “samples” and the 64 widely distributed (in at least seven regions, see Supplementary Dataset S1) Last Glacial species presence/absence (0/1) as “species” for Principal Component Analysis (PCA) and Principal Coordinates Analysis with Bray-Curtis distances (PCoA; CANOCO for Windows v. 5.0). The dataset similarities were also analysed using cluster analysis based on Jaccard similarity index (Past v. 1.88). We also performed Analysis of Similarity (ANOSIM) between chosen groups of regions defined by PCA (Recent Altai–Sayan and Kazakhstan vs. remaining groups as defined by first and second ordination axis, Fig. 1, Table S2). Analysis of Similarity was based on Jaccard similarity index (Past v. 1.88). We show R-values that vary from 0 (complete similarity) to 1 (no similarity) between examined groups. Statistical significance (P) was computed by permutation test (999 permutations i.e. random assignment of faunas to groups).

### Comparison of species presence between Recent and Last Glacial faunas

We compared the proportion of Last Glacial species surviving in the Recent fauna for each of the 14 major areas examined. Only the species positively recorded for Last Glacial in the region in question were counted as either extinct or surviving into the Recent. This approach aimed to solve the problem of the incompleteness of the fossil record that leads to uncertainties whether a species absent in the glacial deposits is a true Holocene immigrant, or has simply not yet been discovered in the fossil record.

### Refugium index

Mere comparison of extinct/surviving species proportions in various regions does not provide sufficiently detailed information on extinction patterns, as the region-specific Last Glacial faunas differed considerably. To determine possible refugia among the examined regions, we used 64 widespread (present in at least seven of 14 regions) Last Glacial species and compared their Recent occurrence in the regions analysed. Values for each species were given as the number of regions in which the species became extinct in the Holocene (see Supplementary Dataset S2 online). The highest value thus obtained was for species with maximum contraction of their range (e.g. saiga antelope *Saiga tatarica*), and the lowest value for species with no contraction of their range (e.g. grey wolf, *Canis lupus*). Sums of the species values for each region were used as a “refugium index” (Ri). To obtain values of refugium index for each species in a given region, the regional refugium index (ri) was divided by the number of widespread Last Glacial species present in the region. The scarcity of Holocene data did not allow us to evaluate continuity of distribution for every species in presumed refugia.

### Species survival rate analysis

In order to analyse geographical patterns more thoroughly, we used 52 “core species”. They were selected according to the criteria as follows: a “core species” was (i) once distributed in at least four regions examined, (ii) displays clear continuity of Pleistocene and Recent distribution in at least one region, and (iii) now survives in at least one region (see Supplementary Dataset S3 online). This selection excluded species whose distribution did not change (i.e. grey wolf *Canis lupus*), species extinct in all examined regions (i.e. muskox *Ovibos moschatus*), species with limited distribution (endemic spp., i.e. Pyrenean ibex *Capra pyrenaica*) and species without continuity of Pleistocene and Recent distribution (i.e. Bactrian camel *Camelus ferus*). The number of extinct/surviving species shared between every pair of regions was counted (Tables 1 and 2). For the analysis of range retraction patterns, we counted how many Last Glacial species extinct in a given region are still present in other examined Recent regions (Table 3). The regions where otherwise extinct species still survive were delimited as Recent “range retraction refugia”.

## Additional Information

**How to cite this article**: Řičánková, V. P. *et al.* Regional patterns of postglacial changes in the Palearctic mammalian diversity indicate retreat to Siberian steppes rather than extinction. *Sci. Rep.* **5**, 12682; doi: 10.1038/srep12682 (2015).

## Supplementary Material

Supplementary Figures and Tables

Supplementary Dataset

## Figures and Tables

**Table 1:**
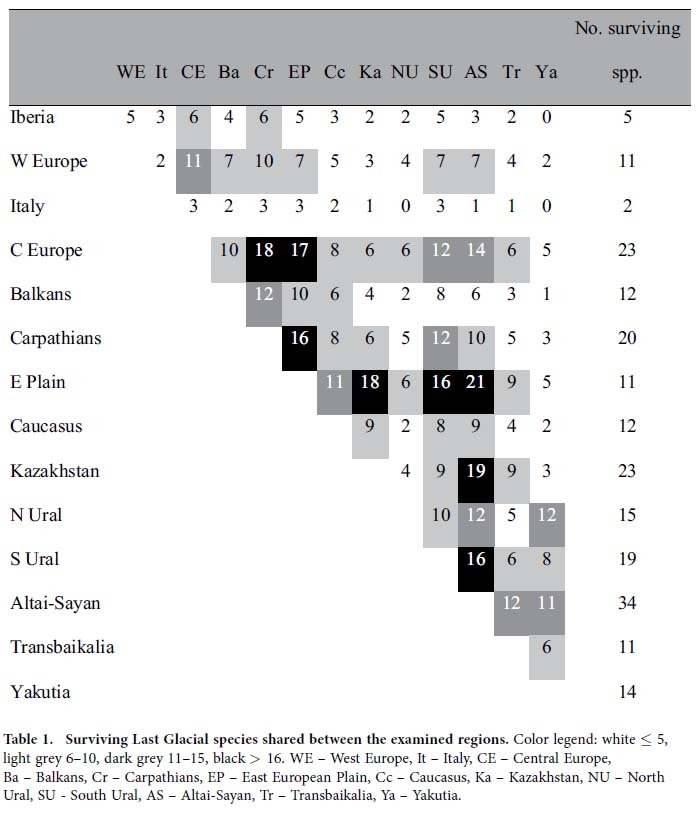
Surviving Last Glacial species shared between the examined regions.

**Table 2:**
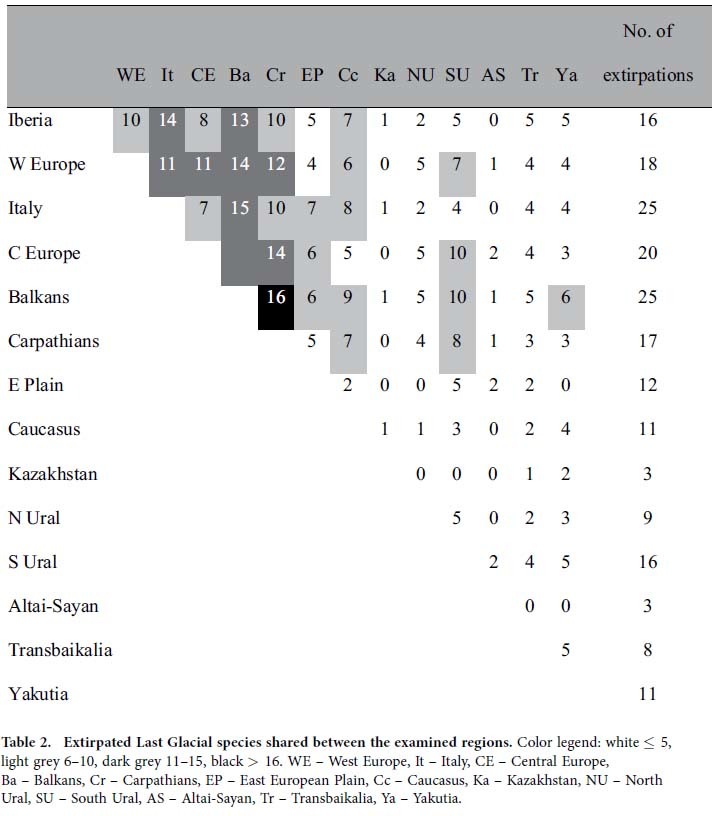
Extirpated Last Glacial species shared between the examined regions.

**Table 3:**
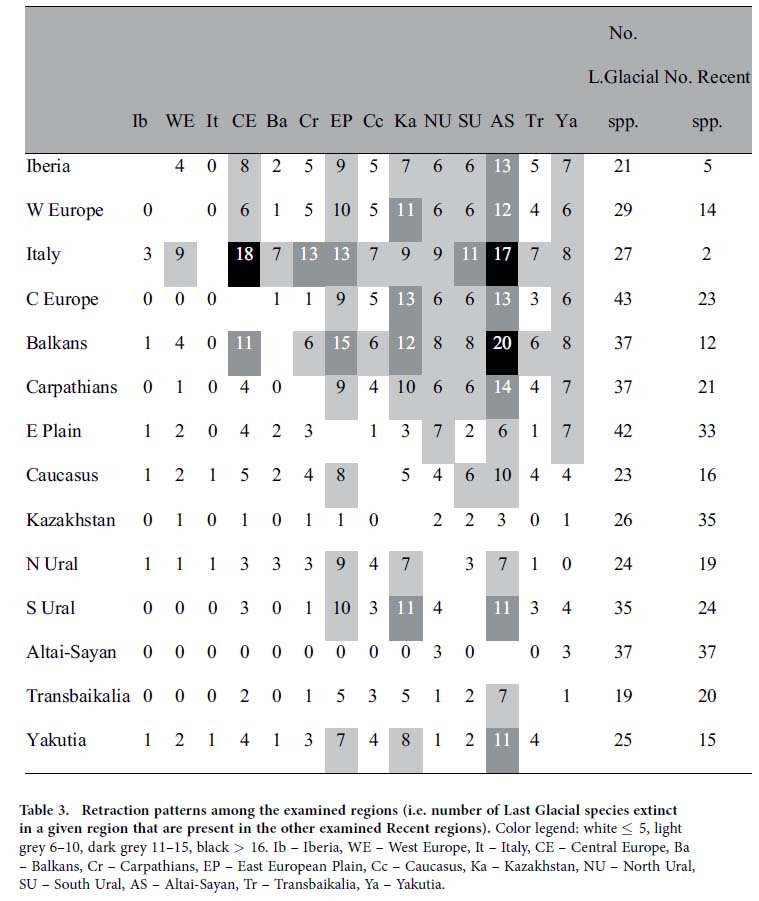
Retraction patterns among the examined regions (i.e. number of Last Glacial species extinct in a given region that are present in the other examined Recent regions).
