# Supplementary material for: Regional patterns of postglacial changes in the Palearctic mammalian diversity indicate retreat to Siberian steppes rather than extinction
Source: Sci Rep. 2015 Aug 6;5:12682. doi: 10.1038/srep12682 (PMC4526850; doi:10.1038/srep12682)
Supplement: Supplementary Figures and Tables [file srep12682-s1.pdf]

Supplementary information

Regional patterns of postglacial changes in the Palearctic mammalian diversity indicate retreat to Siberian steppes rather than extinction

Věra Pavelková Řičánková, Jan Robovský, Jan Riegert and Jan Zrzavý

Figure S1.
Projection scores of studied localities (PcoA with Bray-Curtis distances), according to the presence/absence of the 64 widespread Last Glacial species. We show diagrams for a) sample (faunas) similarities and b) species similarities, points that are close to each other exhibit more similar datasets compared to the others. The first two axes describe 74% of variance. For species abbreviations see electronic supplementary material Dataset S1. *Vulpes vulpes*, *Canis lupus* and *Ursus arctos* were invariably present in all examined regions.

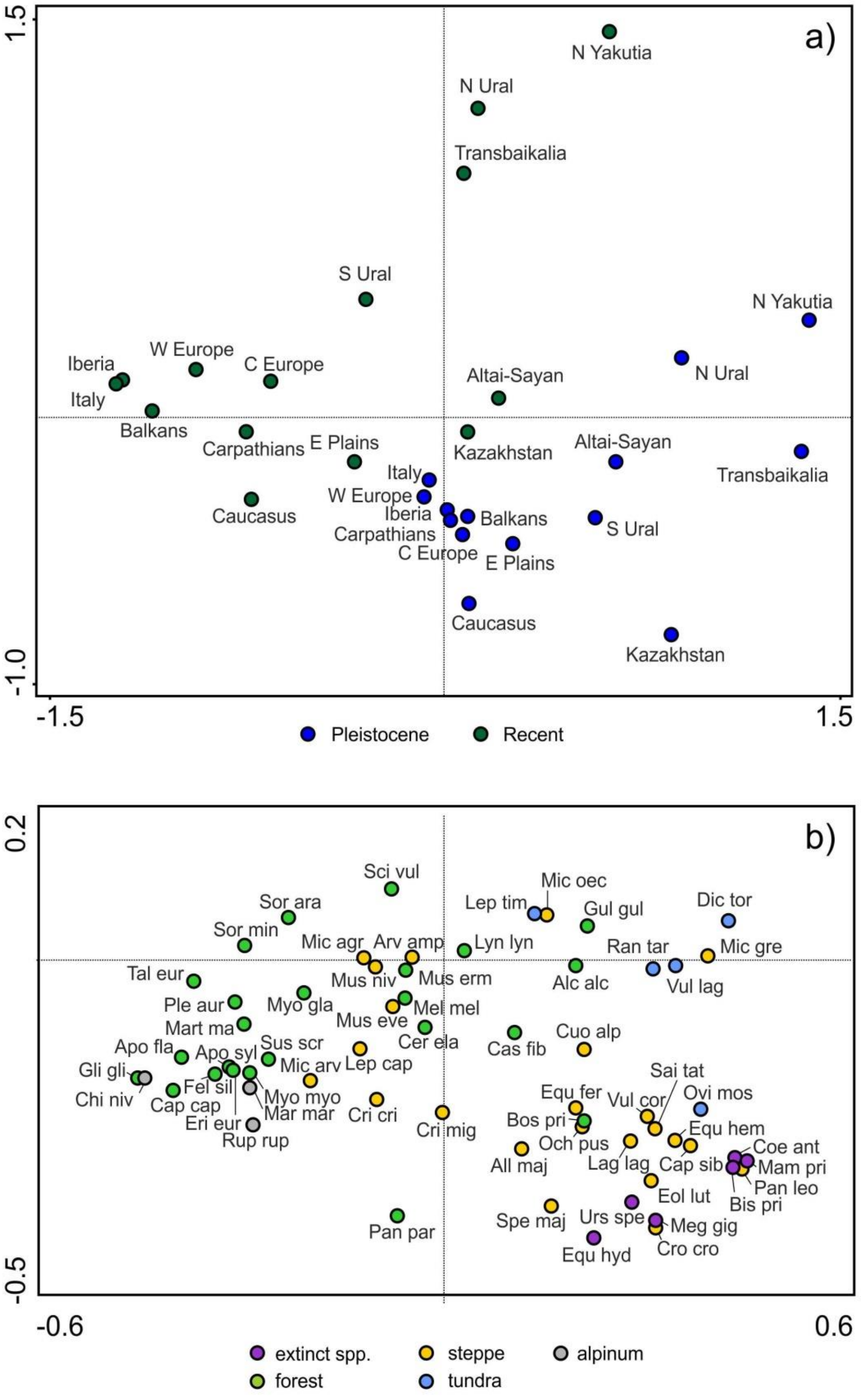

Figure S2.
Cluster analysis based on Jaccard similarity index and single linkage method. Cophenetic correlation coefficient = 0.7793. (R) – Recent, (P) - Pleistocene

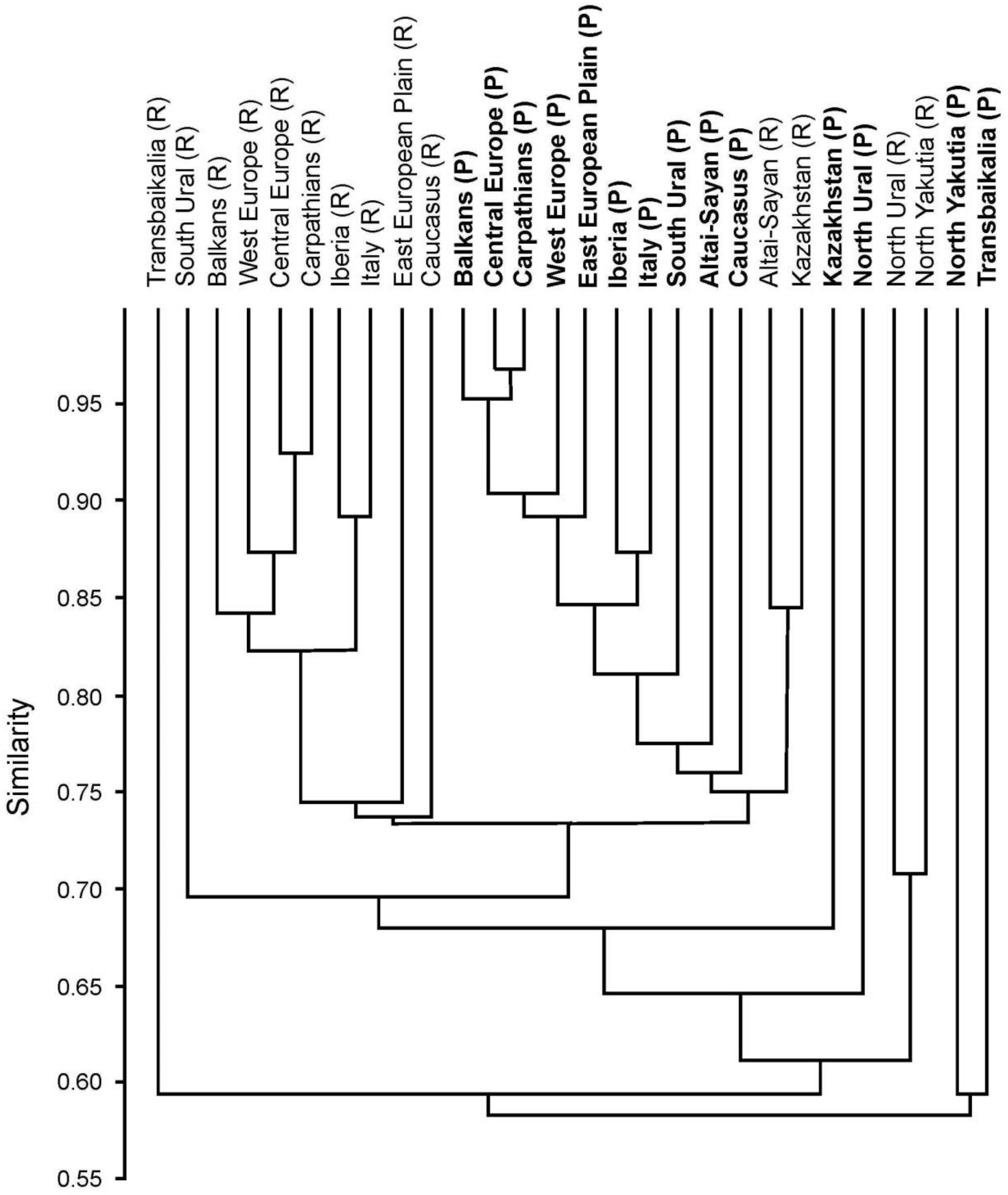

Table S1. Results of Analysis of Similarity for chosen area groups based on Jaccard similarity index.

First group	Second group	R - statistics	P value
Pleistocene AS+Ka+NU+SU+Ya+Tr	Recent AS+Ka	0.31	0.1442
Pleistocene Ba+Cc+Ib+It+EP+WE+CE+Cr	Recent AS+Ka	1.00	0.0290
Recent NU+SU+Ya+Tr	Recent AS+Ka	0.43	0.1339
Recent Ba+Cc+Ib+It+EP+WE+CE+Cr	Recent AS+Ka	0.87	0.0209

Ib – Iberia, WE – West Europe, It – Italy, CE – Central Europe, Ba – Balkans, Cr – Carpathians, EP – East European Plain, Cc – Caucasus, Ka – Kazakhstan, NU – North Ural, SU- South Ural, AS – Altai-Sayan, Tr – Transbaikalia, Ya – Yakutia.

Table S2. Local extirpations**.** Percentage of the extirpated species in the examined Palearctic regions**.**

Region	% extirpated spp.	% extirpated spp. without globally extinct ones
Iberia	43	33
W Europe	39	33
Italy	52	47
C Europe	35	27
Balkans	42	36
Carpathians	33	28
E Plain	33	22
Caucasus	25	17
S Ural	47	38
N Ural	41	33
Kazakhstan	23	10
Altai-Sayan	18	9
Transbaikalia	41	33
Yakutia	43	38

Table S3. Palearctic regions used in the analyses

Region	Country	References
Iberia	Iberian Peninsula - Spain and Portugal	[1-39]
Western Europe	France, Belgium, the Netherlands, Luxembourg	[12, 14-15, 18, 23-27, 29, 32-35, 40-53]
Italy	Italian Peninsula - Italy, exluding Alps	[1-2, 15, 17-18, 20-21, 23, 25-27, 29, 32-35, 38, 54-73]
Central Europe	Central Europe without Carpathians (i.e. Germany, Poland, Austria, West Czech republic)	[14, 17-18, 20-21, 23-27, 29, 32-35, 38, 50, 74-110]
Balkans	Balkan Peninsula - SE Europe south of Drava and Danube Rivers	[1-2, 17-18, 20-21, 23-27, 29, 32-36, 38, 83, 91, 100, 111-127]
Carpathians	Carpathians Mts. (i.e. east Moravia, Slovakia, Hungary, Romania, a part of Ukraine)	[18, 24, 26, 29, 35, 48, 74, 80-82, 85, 89, 91-92, 106, 128-132]
[Southern] East Plains	Russia and Ukraine, southern Belarus, Moldova, westermost Kazakhstan, Dnepr River, Don River, Severskiy Donets River, Volga River basin, Black Sea region, Crimea, 44° to 53°N; 27° to 53°E	[18, 20, 23-25, 29, 32-35, 38, 48, 79, 83, 90, 95, 105, 120, 130, 133-153]
Caucasus	Russia, Armenia, Georgia, Azerbaijan, Turkey, Iran, borders defined according to WWF Caucasus ecoregion: 38° to 45° N; 39° to 50° E	[2, 18, 20, 38, 60, 83, 105, 136, 141, 143, 145, 148, 149, 150, 152, 154-163]
Kazakhstan	Kazakhstan	[18, 20, 38, 79, 120, 141, 149, 164-172]

North Ural	Russia, Ural Mts north of 60° N (i.e. Polar and Northern Urals)	[18, 38, 115, 136, 141, 146, 156, 173-178]
South Ural	Russia, Ural Mts south of 60° N (i.e. Middle and Southern Urals)	[18,38, 115, 120, 136, 141, 144, 146, 156, 173-182]
Altai-Sayan	Russia, Mongolia, China, Kazakhstan, 48° to 54°N; 81° to 108° E	[1, 18, 20, 38, 79, 90, 120, 136, 141, 146, 149, 151, 157-159, 173, 175, 177, 183-209]
Transbaikalia	Russia, Mongolia, 49° to 57°N; 105° to 120°E	[18, 38, 79, 90, 120, 136, 141, 146, 149, 151, 173, 177, 183, 187, 188, 191-195, 200, 203, 209-212]
[Northern] Yakutia	Russia, limited by Lena River W, Kolyma River E, Aldan River S, incl. Cherskiy and Verkhovyanskiy Ridges	[18, 20, 25, 31, 35, 38, 79, 83, 115, 120, 136, 141, 146, 149, 150, 173-174, 177, 185, 199, 211, 213-221]

References

1. Agadjanian, A. K. & Serdyuk, N. V. The history of mammalian communities and paleogeography of the Altai Mountains in the Paleolithic. *Paleontol. J.* **39**, 645-821 (2005).
2. Alberdi, M. T., Caloi, L., Dubrovo, I., Palombo, M. R. & Tsoukala E. Large mammal faunal complexes and palaeoenviromental changes in the late Middle and Late Pleistocene: a preliminary comparison between the Eastern European and the Mediterranean areas. *Geologija* **25**, 8-19 (1998).
3. Altuna, J. Fauna de mamiferos de los yacimentos prehistoricos de Guipuzcoa. Noc catálogo de los mamíferos cuaternairos del Cantábrico y del Pirineo Occidental. *Munibe* **24**, 1-464 (1972).
4. Altuna, J. Restos oseos del yacimiento prehistorico del Rascaňo. *Centro de Investigation y Museo de Altamira, Monografias* **3**, 223-269 (1981).
5. Altuna, J. in *La Riera cave: Stone Age Hunter-Gatherer in Northern Spain* (eds Straus, L. & Clark, G.) 237-274 (Arizona State University, 1986).
6. Altuna, J., Mariezkurrena, K. & Elorza, M. Arquezoología de los niveles paleolíticos de la Cueva de Abauntz (Arraiz, Navarra). *Saldvie* **2**, 1-26 (2001-2002).
7. Altuna, J. & Mariezkurrena, K. Bases de subsistencia de los pobladores de Erralla: macromamíferos. *Munibe* **37**, 87-117 (1985).
8. Altuna, J. & Straus, L. G. The solutrean of Altamira: the artifactual and faunal evidence. *Zephyrus* **26/27**, 175-182 (1976).

9. Álvarez-Lao, D. & García, N. Geograpical distribution of Pleistocene cold-adapted large mammal faunas in the Iberian Peninsula. *Quat. Int.* **233**, 159-170 (2011).
10. Arsuaga, J. L. *et al.* New Neanderthal remains from Cova Negra (Valencia, Spain). *J. Hum. Evol.* **52**, 31-58 (2007).
11. Benito, C. Paleoclimatical interpretation of the Quaternary small mammals of Spain. *Geobios* **27**, 753-767 (1994).
12. Brunet-Lecomte, P. La répartition géographique des campagnols souterrains (Arvicolidae, Rodentia) au Quaternaire en Europe occidentale. *Mammalia* **53**, 605-619 (1989).
13. Cardoso, J. L. Les grands mammifères du pléistocène supérieur du Portugal. Essai de synthese. *Geobios* **29**, 235-250 (1996).
14. Crégut-Bonnoure, E. Dynamics of bovid migration in Western Europe during the Middle and Late Pleistocene. *Cour. Forsch.-Inst. Senckenberg* **153**, 177-185 (1992).
15. Cuenca-Bescós, G. *et al.* Late Quaternary small mammal turnover in the Cantabrian Region: The extinction of *Pliomys lenki* (Rodentia, Mammalia). *Quat. Int.* **212**, 129-136 (2010).
16. Davis, S. J. M. The mammals and birds from the Gruta do Caldeirão, Portugal. *Revista Portuguesa de Arquelogia* **5**, 29-98 (2002).
17. Fedosenko, A. K. & Blank, D. A. *Capra sibirica*. *Mamm. Species* **675**, 1-13 (2001).
18. Finlayson, C. & Carrión, J. S. Rapid ecological turnover and its impact on Neanderthal and other human populations. *Trends Ecol. Evol.* **22**, 213-222 (2007).
19. García, N. & Arsuaga, J. L. Last Glaciation cold-adapted faunas in the Iberian Peninsula. *Deinsea* **9**, 159-169 (2003).
20. Groves, C. P. *Horses, Asses and Zebras in the Wild* (Davis and Charles, 1974).
21. Hockett, B. & Haws, J. A Taphonomic and methodological perspectives of leporid hunting during the Upper Paleolithic of the Western Mediterranean Basin. *J. Archaeol. Method. Th.* **9**, 269-302 (2002).
22. Jennings, R. P. *et al.* New dates and palaeoenvironmental evidence for the Middle to Upper Palaeolithic occupation of Higueral de Valleja Cave, southern Spain. *Quat. Sci. Rev.* **28**, 830-839 (2009).
23. Kahlke, R. D. Repeated immigration of saiga into Europe. *Cour. Forsch.-Inst. Senckenberg* **153**, 187-195 (1992).
24. Kowalski, K. Pleistocene rodents of Europe. *Folia Quaternaria* **72**, 3-389 (2001).
25. Kurtén, B. *Pleistocene Mammals of Europe* (Weidenfeld and Nicolson, 1968).
26. Markova, A. & Puzachenko, A. in *Evolution of European Ecosystems during Pleistocene- Holocene Transition (24-8 kyr BP)* (eds Markova, A. K. *et al.*) Ch. 4, 117-160 (KMK Scientific Press, 2008).
27. Mitchell-Jones, A. J. *et al. The Atlas of European Mammals* (Academic Press, 1999).
28. Peman, E. Aspectos climáticos y ecológicos de los micromamíferos del yacimiento

de Erralla. *Munibe* **37**, 49-57 (1985).

29. Raufussl, I. & von Koenigswald, W. New remains of Pleistocene *Ovibos moschatus* from Germany and its geographic and stratigraphic occurrence in Europe. *Geologie en Mijnbouw* **78**, 383-394 (1999).
30. Rivals, F., Schulz, E. & Kaiser, T. M. Late and middle Pleistocene ungulates dietary diversity in Western Europe indicate variations of Neanderthal paleoenviromental through time and space. *Quat. Sci. Rev.* **28**, 3388-3400 (2009).
31. Sher, A. W. Säugetierfunde und Pleistozänstratigraphie in der Kolyma-Niederung. *Ber. Dtsch. Ges. Geol. Wiss. Reihe A* **16**, 113-125 (1971).
32. Sommer, R. & Benecke, N. Late- and Post-glacial history of the Mustelidae in Europe. *Mamm. Rev.* **34**, 249-284 (2004).
33. Sommer, R. & Benecke, N. Late-Pleistocene and early Holocene history of the canid fauna of Europe (Canidae). *Mamm. Biol.* **70**, 227-241 (2005).
34. Sommer, R. & Benecke, N. Late Pleistocene and Holocene development of the felid fauna (Felidae) of Europe: a review. *J. Zool.* **269**, 7-19 (2006).
35. Sommer, R. S. & Nadachowski, A. Glacial refugia of mammals in Europe: evidence from fossil record. *Mamm. Rev.* **36**, 251-265 (2006).
36. Straus, L. G. *et al.* Paleoecology at La Riera (Austrias, Spain). *Curr. Anthropol.* **22**, 655-682 (1981).
37. Straus, L. G. & Clark, G. A. La Riera Cave. Stone age hunter-gatherer adaptations in northern Spain. *Anthropological Research Papers* (*Arizona State Univ.*) **36**, 1-502 (1986).
38. Wilson, D. E. & Reeder, D.-A. M. *Mammal Species of the World. A Taxonomic and Geographic Reference* (Johns Hopkins University Press, 2005).
39. Zilhão, J. Nature and culture in Portugal from 30 000 to 20 000 BP. *Analecta Praehistorica Leidensia* **31**, 337-354 (1999).
40. Aguilar, J. P., Pélissié, T., Sigé, B. & Michaux, J. Occurrence of the Stripe Field Mouse lineage (*Apodemus agrarius* Pallas 1771; Rodentia; Mammalia) in the Late Pleistocene of southwestern France. *C. R. Palevol.* **7**, 217-225 (2008).
41. Auguste, P. Évolution des peuplements mammaliens en Europe du nord-ouest durant le pléstocène moyen et supérieur. Le cas de la France septerntrionale. *Quaternaire* **20**, 527-550 (2009).
42. Chaline, J., Brunet-Lecomte, P. & Campy, M. The last glacial/interglacial record of rodent remains from the Gigny karst sequence in the French Jura used for palaeoclimatic and palaeoecological reconstructions. *Palaeogeogr. Palaeoclimatol. Palaeoecol.* **117**, 229-352 (1995).
43. Clot, A. & Evin, J. Gisements naturels Pléistocenes et Holocènes des cavités des Pyrénées Occidentales Françaises: Inventaire et datages 14 C. *Munibe* **38**, 185-194 (2008).
44. Crochet, J. Y, Gence, J., Boulbes, N., Boutié, P. & Cretin, C. Nouvelles données paléoenvironnementales dans le Sud de la France vers 30000 ans 14C BP: le cas de la grotte Marie (Hérault). *C. R. Palevol*. **6**, 241-251 (2007).
45. Magniez, P. Nouvelles donneés sur le genre *Capra* Linné, 1758 (Mammalia,

Bovidae) du pléistocène supérieur de la grotte Tournal (Bize-Minervois, France): implications biochronologiques et évolutives. *Quaternaire* **20**, 509-525 (2009).

46. Montuire, S. & Desclaux, E. Palaeoecological analysis of mammalian faunas and enviromental evolution in the South of France during the Pleistocene. *Boreas* **26**, 355-365 (1997).
47. Montuire, S., Michaux, J., Legendre, S. & Aguilar, J. P. Rodents and climate. 1. A model for estimating past temperatures using arvicolids (Mammalia: Rodentia). *Palaeogeogr. Palaeoclimatol. Palaeoecol.* **128**, 187-206 (1997).
48. Musil, R. Paleobiography of terrestrial communities in Europe during the Last glacial. *Acta Musei Nationalis Pragae* **41**, 1-84 (1985).
49. Reumer, J. W. F. Quaternary Insectivora (Mammalia) from southwestern France. *Acta Zool. Cracov.* **39**, 413-426 (1996).
50. Schmölcke, U. & Zachos, F. E. Holocene distribution of the moose (*Alces alces*, Cervidae) in Central Europe. *Mamm. Biol.* **70**, 329-344 (2005).
51. Sevilla, P. & Chaline, J. New data on bat fossils from Middle and Upper Pleistocene localities of France. *Geobios* **44**, 289-297 (2011).
52. Van Kolfschoten, T. Pleistocene mammals from the Netherlands. *Boll. Soc. Paleontol. I.* **40**, 209-215 (2001).
53. Volf, J. *Bovids* (SZN, 1987).
54. Abbassi, M. & Brunet-Lecomte, P. *Terricola* Fatio 1867 (Arvicolidae, Rodentia) de cinq séquences du Sud-Est de la France et de Ligurie. *Quaternaire* **8**, 3-12 (1997).
55. Barbato, L. C. & Gliozzi, E. Late Pleistocene micromammal association from Praia a Mare (Calabria, Southern Italy): palaeoclimatological and biochronological implications. *Boll. Soc. Paleontol. I.* **40**, 159-166 (2001).
56. Basset, P., Yannic, G. & Hausser, J. Genetic and karyotypic structure in the shrews of the *Sorex araneus* group: are they independent? *Mol. Ecol.* **15**, 1577-1587 (2006).
57. Bona, F., Laurenti, B. & Delfino, M. Climatic fluctuations during the Last Glacial in the North-Western Lombardian Prealps: the Upper Pleistocene faunal assemblages of the Caverna Generosa (Como, Italy). *Riv. Ital. Paleontol. S.* **115**, 253-267 (2009).
58. Capasso-Barbato, L. & Gliozzi, E. Late Pleistocene micromammal association from Praia a Mare (Calabria, Southern Italy): Palaeoclimatological and biochronological implications. *Boll. Soc. Paleontol. I.* **40**, 159-166 (2001).
59. Cassoli, P. F. & Tagliacozzo, A. I macromammiferi dei livelli tardopleistocenici delle Arene Candide (Savono, Italia): considerazioni paleontologiche e archeozoologiche. *Quaternaria Nova* **4**, 101-262 (1994).
60. Palma di Cesnola, A. Le Paléolithique supérieur en Italie. *Collection L´homme des origines, série Prehístoire d´Europe* **9**, 1-482 (2001).
61. Desclaux, E., Abbassi, M., Marquet, J-C., Chaline, J. & van Kolfschoten, T. Distribution and evolution of *Arvicola* Lacépède, 1799 (Mammalia, Rodentia) in France and Liguria (Italy) during the Middle and Upper Pleistocene. *Acta Zool. Cracov.* **43**, 107-125 (2000).

62. Ferraris, M., Sala, B. & Scola, V. The Late Pleistocene fauna with *Pliomys lenki* from the Ghiacciaia Cave loess (Northern Italy). *Quat. Int.* **5**, 71-79 (1990).
63. Kaniewski, D., Renault-Miskovsky, J. & de Lumley, H. Palaeovegetation from a *Homo neanderthalensis* occupation in Western Liguria: archaeopalynology of Madonna dell´Arma (San Remo, Italy). *J. Archaeol. Sci.* **32**, 827-840 (2005).
64. Marra, A. C. Pleistocene mammal faunas of Calabria (Southern Italy): biochronology and palaeobiogeography. *Boll. Soc. Paleontol. I.* **48**, 113-122 (2009).
65. Mazza, P., Rustioni, M., Agostini, S. & Rossi, A. An unexpected Late Pleistocene macaque remain from Grotta degli OrsiVolanti (Rapino, Chieti, central Italy). *Quat. Int.* **212**, 149-158 (2010).
66. Palombo, M. R. Climate change versus biotic interaction: a case study of large mammal faunal complexes on the Italian Peninsula from the Pliocene to the Late Pleistocene. New methodological approaches. *Cour. Forsch.-Inst. Senckenberg* **259**, 13-46 (2007).
67. Petronio, C. *et al.* The Late Pleistocene fauna from Ingarano (Gargano, Italy): biochronological, palaeoecological, paleoethnological and geochronological implications. *B. Soc. Ital.* **34**, 333-339 (1996).
68. Petronio, C., Di Canzio, E. & Salari, L. The Late Pleistocene and Holocene mammals in Italy: new biochronological and paleoenviromental data. *Palaeontographica A* **279**, 147-157 (2007).
69. Rustioni, M., Ferretti, M. P., Mazza, P., Pavia, M. & Varola, A. The vertebrate fauana from Cardamone (Apulia, southern Italy): an example of Mediterranean mammoth fauna. *Deinsea* **9**, 395-403 (2003).
70. Sala, B. Variations climatiques et séquences chronologiques sur la base des variations des associations fauniques à grands mammifèrs. *Rivista di Scienze Preistoriche* **38**, 161-180 (1983).
71. Salari, L. & Sardella, R. The Pleistocene porcupine *Hystrix vinogradovi* Argyropulo, 1941 in Italy. *Boll. Soc. Paleontol. I.* **48**, 123-127 (2009).
72. Valensi, P. Évolution des peuplement de grands mammifès en Europe Méditerranéenne occidentale durant le Pléistocène moyen et supérieur. Un exemple régional: les Alpes du sud Francaises et Italiennes. *Quaternaire* **20**, 551-567 (2009).
73. Valensi, P. & Psathi E. Faunal Exploitation during the Middle Palaeolithic in South-eastern France and North-western Italy. *Int. J. Osteoarchaeol.* **14**, 256-272 (2004).
74. Anděra, M. & Gaisler, J. *Mammals of the Czech Republic* (Academia, 2012).
75. Benda, P., Ruedi, M. & Uhrin, M. First record of *Myotis alcathoe* (Chiroptera: Vespertillionidae) in Slovakia. *Folia Zool. Brno* **52**, 359-365 (2003).
76. Cyrek, K. *et al.* Excavation in the Deszczowa cave (Kroczyckie rocks, Częstochowa Upland, Central Poland). *Folia Quaternaria* **71**, 5-84 (2000).
77. Harrison, D. L. Systematic status of Kennard’s Shrew (*Sorex kennardi* Hinton, 1911, Insectivora: Soricidae): a study based on British and Polish material. *Acta*

Zool. Cracov. **39**, 201-212 (1996).

78. Heinrich, W. D. Erster Nachweis von *Lagurus lagurus* (Pallas, 1773) (Mammalia, Rodentia, Arvicolidae) für das Jungpleistozän Nordeutschlands. *Lynx* **32**, 89-96, (2001).
79. Heptner, V. G. *et al. Die Säugetiere der Sowjetunion, Band II: Seekühe und Raubtiere* (VEB Gustav Fischer Verlag, 1974).
80. Horáček, I. Fossil record and chorological status of dormice in Czechoslovakia. Part I. *Glis glis, Eliomys quercinus*. *Folia Mus. Rer. Natur. Bohem. Occid. Plzeň Zoologica* **24**, 49-59 (1986).
81. Horáček, I. & Marco, A. S. Comments on the Weichselian small mammal assemblages in Czechoslovakia and their stratigraphical interpretation. *Neues Jahrb. Geol. Paläontol.* **9**, 560-576 (1984).
82. Horáček, I. & Ložek, V. Palaeozoology and the Mid-European Quaternary past: scope of the approach and selected results. *Rozpravy ČSAV, řada matematických a přírodních věd* **98**, 1-102 (1988).
83. Horáček, I., Hanák, V. & Gaisler, J. Bats of the Palearctic region: a taxonomic and biogeographic review. *Proceedings of the VIIIth EBRS* **1**, 11-157 (2000).
84. Horáček, I., Ložek, V., Svoboda, J. & Šajnerová, A. Nature and human setlement of the karst in the Late Paleolite and Mesolite. *The Dolní Věstonice studies* **7**, 313-343 (2002).
85. Jánossy, D. *Pleistocene Vertebrate Faunas of Hungary* (Elsevier, 1986).
86. Kalthoff, D. C., Mörs, T. & Tesakov, A. Late Pleistocene small mammals from the Wannenköpfe volcanoes (Neuwied Basin, western Germany) with remarks on the stratigraphic range of *Arvicola terrestris*. *Geobios* **40**, 609-623 (2007).
87. Kowalski, K. History and evolution of the terrestrial fauna of Poland. *Folia Quaternaria* **59/60**, 1-389 (1989).
88. Kowalski, K. Lemmings (Mammalia, Rodentia) as indicators of temperature and humidity in the European Quaternary. *Acta Zool. Cracov.* **38**, 85-94 (1995).
89. Krištofík, J. & Danko, Š. *Mammals of Slovakia - Distribution, Bionomics and Conservation* (Veda, 2012).
90. Markova, A. K., Puzachenko, A. Yu. & van Kolfschoten, T. The North Eurasian mammal assemblages during the end of MIS 3 (Brianskian-Late Karginian-Denekamp Interstadial). *Quat. Int.* **212**, 149-158 (2010).
91. Musil, R. in *Neanderthals and Modern Humans in the European Landscape during the Last Glaciation* (eds van Andel, T. H. & Davies, W.) Ch. 10, 167-190 (McDonald Institute for Archaeological Reserach, 2004).
92. Musil, R. The enviroment of the Middle Palaeolithic sites in Central and Eastern Europe. *Acta Univ. Wratislav.* **3207**, 1-59 (2010).
93. Münzel, S. C. Subsistence patterns in the Gravettien of the Ach Valley, a former tributary of the Danube valley in the Swabian Jura. *The Dolní Věstonice Studies* **11**, 71-85 (2004).

94. Münzel, S. C. & Conard, N. J. Change and Continuity in Subsistence during the Middle and Upper Palaeolithic in the Ach Valley of Swabia (South-west Germany). *Int. J. Osteoarchaeol.* **14**, 225-243 (2004).

95. Nadachowski, A. *Late Quaternary Rodents of Poland with Special Reference to Morphotype Dentition Analysis of Voles* (Polska Akademia Nauk, 1982).

96. Nadachowski, A. Review of fossil Rodentia from Poland. *Senckenb. Biol.* **70**, 229-250 (1989).

97. Nadachowski, A., Harrison, D. L., Szyndlar, Z., Tomek, T. & Wolsan, M. Late Pleistocene vertebrate fauna from Obłazowa 2 (Carpathians, Poland): palaeoecological reconstruction. *Acta Zool. Cracov.* **36**, 281-290 (1993).

98. Nadachowski, A. *et al. Late Pleistocene Environment of the Częstochowa Upland (Poland) Reconstructed on the Basis of Faunistic Evidence from Archaeological Cave Sites* (Institute of Systematics and Evolution of Animals, Polish Academy of Sciences, 2009).

99. Ochman, K. Late Pleistocene and Holocene bats (Chiroptera) fom the Komarowa Cave (Cracow-Częstochowa Upland, Poland) - preliminary results. *Acta Zool. Cracov.* **46**,73-84 (2003).

100. Petculescu, A. & Ştiucă, E. Peculiarity of the mammal associations from the Upper Pleistocene (Dobrogea, Romania). *Quat. Int.* **179**, 79-82 (2008).

101. Rofes, J. *et al.* The southwesternmost record of *Sicista* (Mammalia; Dipodidae) in Eurasia, with a re-view of the palaeogeography and palaeoecology of the genus in Europe. *Palaeogeogr. Palaeoclimatol. Palaeoecol.* **348-349**, 67-73 (2012).

102. Rzebik-Kowalska, B. Climate and history of European shrews (family Soricidae). *Acta Zool. Cracov.* **38**, 95-107 (1995).

103. Rzebik-Kowalska, B. Erinaceomorpha and Soricomorpha (Mammalia) from the Late Pleistocene and Holocene of Krucza Skała Rock Shelter and Komarowa Cave (Poland). *Acta Zool. Cracov.* **49**, 83-118 (2006).

104. Rzebik-Kowalska, B. *Biodiversity of Polish Fossil Insectivores (Erinaceomorpha, Soricomorpha, Insectivora, Mammalia) Compared to the European and Global Faunas* (Institute of Systematics and Evolution of Animals, Polish Academy of Sciences, 2009).

105. Smith, F. H., Janković, I. & Karavanić, I. The assimilation model, modern human origins in Europe, and the extinction of Neanderthals. *Quat. Int.* **137**, 7-19 (2005).

106. Spitzenberger, F. *Die Säugetierfauna Österreichs* (Grüne Reihe des Bundesministeriums für Land- und Forstwirtschaft, Umwelt und Wasserwirtschaft, 2001).

107. Storch, G. Local differentiation of faunal change at the Pleistocene-Holocene boundary. *Cour. Forsch.-Inst. Senckenberg* **153**, 135-142 (1992).

108. Street, M. & Baales, M. in *The Holocene History of the European Vertebrate Fauna. Modern Aspects of Research* (ed. Benecke, N.) Ch. 2, 9-38 (Verlag Marie Leidorf GmbH, 1999).

109. Woloszyn, B. W. Pliocene and Pleistocene bats of Poland. *Acta Palaeontol. Pol.* **32**, 207-325 (1987).

110. Zimina, R. P. & Gerasimov, I. P. The Periglacial expansion of marmots (*Marmota*) in Middle Europe during Late Pleistocene. *J. Mammal.* **54**, 327-340 (1973).

111. Bailey, G. N., Carter, P. L., Gamble, C. S. & Higgs, H. P. Asprochaliko and Kastritsa: further investigations of Palaeolithic settlement and economy in Epirus (North-West Greece). *Proc. Prehist. Soc.* **49**, 15-42 (1983).

112. Brunet-Lecomte, P., Nadachowski, A. & Chaline, J. *Microtus* (*Terricola*) *grafi* nov. sp. du Pléistocène supérieur de la grotte de Bacho Kiro (Bulgarie). *Geobios* **25**, 505-509 (1992).

113. Chatzopoulou, K. The Late Pleistocene small mammal fauna from the Loutra Aridea bear-cave (Pella, Macedonia, Greece) - additional data. *Atti. Mus. Civ. Stor. Nat. Trieste* **49**, 35-45 (2003).

114. Dimitrijević, V. Upper Pleistocene mammals from cave deposits in Serbia. *Acta Zool. Cracov.* **39**, 117-120 (1996).

115. Flint, V. E., Čugunov, JuD. & Smirin, V. M. *Mammals of USSR* (Izdatelstvo Mysl, 1965).

116. Harvati, K., Panagopoulou, E. & Karkanas, P. First Neanderthal remains from Greece: the evidence from Lakonis. *J. Hum. Evol.* **45**, 465-473 (2003).

117. Kozlowski, J. K. *Excavation in the Bacho Kiro Cave* (*Bulgaria)* (Polish Scientific Publishers, 1982).

118. Malez, M. Die quartären Vertebraten-Faunen in der SFR Jugoslawien. *Quartärpaläontologie* **6**, 101-117 (1986).

119. Montuire, S. & Brunet-Lecomte, P. Relation between climatic fluctuation and morphological variability in *Microtus* (*Terricola*) *grafi* (Arvicolinae, Rodentia) from Bacho Kiro (Bulgaria, Upper Pleistocene). *Lethaia* **37**, 71-78 (2004).

120. Panteleyev, P. A. *The Rodents of the Palaearctic, Composition and Areas* (Russian Academy of Sciences, 1998).

121. Pohar, V. Great mammals descending from the culmination point of the last Glacial in Slovenia. *Razprave IV. Razreda Sazu* **35**, 85-100 (1994).

122. Popov, V. V. Small mammals (Mammalia - Insectivora, Rodentia, Lagomorpha) from Late Pleistocene deposits in Mecha Dupka Cave (the Western Balkan Mountain). I. Taphonomy, paleoecological and zoogeographical peculiarities of the fauna. *Acta Zool. Bulg.* **24**, 35-44 (1984).

123. Popov, V. V. Small mammals (Mammalia - Insectivora, Rodentia, Lagomorpha) from Late Pleistocene deposits in Mecha Dupka Cave (the Western Balkan Mountain). II. Description of species. *Acta Zool. Bulg.* **26**, 23-49 (1985).

124. Popov, V. V. & Marinska, M. An almost one million year long (Early to Late Pleistocene) small mammal succession from the archaeological layers of Kozarnika Cave in Northern Bulgaria. *Cour. Forsch.-Inst. Senckenberg* **259**, 79-92 (2007).

125. Spassov, N. & Raychev, D. Late Wurm *Panthera pardus* remains from Bulgaria: the European fossil leopards and the question of the probable species survival until the Holocene on the Balkans. *Historia Naturalis Bulgarica* **7**, 71-96 (1997).

126. Tsoukala, E. Quaternary faunas of Greece. *Cour. Forsch.-Inst. Senckenberg* **153**, 79-92 (1992).

127. Tsoukala, E. *et al.* Paleontological and stratigraphical research in Loutra Arideas bear cave. *Scientific Annals, School of Geology Aristotle University of Thessaloniki (AUTH)* **98**, 41-67 (2006).

128. Brunet Lecomte, P. & Paunesco, A. C. Morphometrie comparee de la premiere molaire inferieure des campagnols du genre *Microtus* sous-genre *Terricola* dans les carpates roumaines au pleistocene superieur et a l´holocene Rodentia, Arvicolidae. *Ann. Mus. Civ. Stor. Nat. Giacomo Doria* **96**, 567-579 (2004).

129. Horáček, I. Glacial cycles and mammalian biodiversity of Central Europe: large scale migrations or vicariance dynamics. *GeoLines* **11**, 103-107 (2000).

130. Krakhmalnaya, T. V. in *The Holocene History of the European Vertebrate Fauna. Modern Aspects of Research* (ed. Benecke, N.) Ch. 18, 223-235 (Verlag Marie Leidorf GmbH, 1999).

131. Pazonyi, P. Mammalian ecosystem dynamics in the Carpathian Basin during the last 27 000 years. *Palaeogeogr. Palaeoclimatol. Palaeoecol.* **212**, 295-314 (2004).

132. Bihari, Z., Csorba, G. & Heltai, M. *Magyarország Emlőseinek Atlasza. Kossuth Természettár* (Kossuth Kiadó, 2007).

133. Alekseeva, L. I. *Mammals of the Late Pleistocene of the Eastern Europe* (Nauka, 1990).

134. Averianov, A. Pleistocene lagomorphs of Eurasia. *Deinsea* **8**, 1-13 (2001).

135. Benecke, N. in *The Holocene History of the European Vertebrate Fauna. Modern Aspects of Research* (ed. Benecke, N.) Ch. 4, 43-57 (Verlag Marie Leidorf GmbH, 1999).

136. Bobrinskii, N. A., Kuznecov, B. A. & Kuzyakin, A. P. *Guide to the Mammals of the USSR* (Prosveshcheni, 1965).

137. Borziac, I. A. *et al.* The Upper Palaeolithic site of Ciuntu on the Middle Pruth, Moldova: a multidisciplinary study and reinterpretation. *Proc. Prehist. Soc.* **63**, 285-301 (1997).

138. David, A. I. in *The Holocene History of the European Vertebrate Fauna. Modern Aspects of Research* (ed. Benecke, N.) Ch. 5, 59-72 (Verlag Marie Leidorf GmbH, 1999).

139. David, A., Nadachowski, A., Pascaru, V., Wojtal, P. & Borziac, I. Late Pleistocene fauna from the Late Palaeolithic butchering site Cosăuţi 1, Moldova. *Acta Zool. Cracov.* **46**, 85-96 (2003).

140. Markova, A. K. in *Late Quaternary Environments of the Soviet Union* (eds Velichko, A. A., Wright, H. E. Jr. & Barnosky, C. W.) Ch. 20, 209-218 (Longman, 1984).

141. Markova, A. K., *et al.* Late Pleistocene distribution and diversity of mammals in Northern Eurasia (PALEOFAUNA database). *Paleontologia i Evolución* **28-29**, 5-143 (1995).

142. Markova, A. & Puzachenko, A. in *Evolution of European Ecosystems during Pleistocene-Holocene Transition (24-8 kyr BP)* (eds Markova, A. K. *et al.*) Ch. 4.1,

91-116 (KMK Scientific Press, 2008).

143. Markova, A. & Puzachenko, A. in *Evolution of European Ecosystems during Pleistocene-Holocene Transition (24-8 kyr BP)* (eds Markova, A. K. *et al.*) Ch. 4.2, 117-160 (KMK Scientific Press, 2008).

144. Motuzko, A. N. & Navichkova, N. A. *The mammoth and mammoth fauna of Belarus - 3rd International mammoth conference* (2003) Date of access:10/02/2015

145. Orlova, L. A., Kuzmin, Y. V. & Dementiev, V. N. A review of the evidence for extinction chronologies for five species of Upper Pleistocene megafauna in Siberia. *Radiocarbon* **46**, 301-314 (2004).

146. Rekovets, L. I. Periglacial micromammal faunas from the Late Pleistocene of Ukraine. *Acta Zool. Cracov.* **38**, 129-138 (1995).

147. Rieger, I. *Hyaena hyaena*. *Mamm Species* **150**, 1-5 (1981).

148. Sokolov, I. I. *Fauna of USSR, Mammals, Ungulates (Perissodactyla and Artiodactyla)* (Izdatelstvo Akademii Nauk SSSR, 1959).

149. Sunquist, M. & Sunquist, F. *Wild Cats of the World.* (Univ. of Chicago Press, 2002).

150. Titov, V. V. Habitat conditions for *Camelus knoblochi* and factors in its extinction. *Quat. Int.* **179**, 120-125 (2008).

151. Vereshchagin, N. K. *Mammals of Caucasus (History of Faunal Development)* (Izdatelstvo Akademii Nauk SSSR, 1959).

152. Zagorodnyuk, I. Field key to small mammals of Ukraine. *Proceedings of the Theriological School* **5**, 1-60 (2002).

153. Baryshnikov, G.. F. Local biochronology of Middle and Late Pleistocene mammals from the Caucasus. *Russ. J. Theriol.* **1**, 61-67 (2002).

154. Baryshnikov, G. F. Pleistocene Canidae (Mammalia: Carnivora) from the Paleolithic Kudaro caves in the Caucasus. *Russ. J. Theriol.* **11**, 77-120 (2012).

155. Görner, M. & Hackenthal, H. *Säugetiere Europas* (Neumann Verlag, 1987).

156. Matveev, V. A., Kruskop, S. V. & Kramenov, D. A. Revalidation of *Myotis petax* Hollister, 1912 and its new status in connection with *M. daubentonii* (Kuhl, 1817) (Vespertilionidae, Chiroptera). *Acta Chiropt.* **7**, 23-37 (2005).

157. Mazák, V. *Panthera tigris*. *Mamm. Species* **152**, 1-8 (1981).

158. Rossina V. V., Baryshnikov G. F. & Woloszyn B. W. Dynamics of the Pleistocene bat fauna from Matuzka Paleolithic site (Northern Caucasus, Russia) (Chiroptera). *Lynx* **37**, 229-240 (2006).

159. Sokolov, V. E. *Saiga tatarica*. *Mamm. Species* **38**, 1-4 (1974).

160. Thulin, C-G. The distribution of mountain hares *Lepus timidus* in Europe: a challenge from brown hares *L. europaeus*? *Mamm. Rev.* **33**, 29-42 (2003).

161. Vereschagin, N. K. & Baryshnikov, G. F. in *Quaternary Extinctions: A Prehistoric Revolution* (eds Martin, P. S. & Klein R. G.) Ch. 22, 483-516 (Arizona University Press, 1984).

162. Zaitsev, M. V. & Baryshnikov, G. F. Pleistocene Soricidae (Lipotyphla,

Insectivora, Mammalia) from Treugolnaya Cave, Northern Caucasus, Russia. *Acta Zool. Cracov.* **45**, 283-305 (2002).

163. Bazhanov, V. S. & Kostenko, N. N. *Atlas of the Principal Mammal Forms of the Kazakhstan Quaternary* (KAN, 1962).

164. Benda, P. & Reiter, A. On the occurrence of *Eptesicus bobrinskoi* in the Middle East (Chiroptera: Vespertilionidae). *Lynx* **37**, 23-44 (2006).

165. Chlachula, J. Pleistocene climate change, natural enviroments and Palaeolithic occupation of East Kazakhstan. *Quat. Int.* **220**, 64-87 (2010).

166. Gromov, I. M. & Erbajeva, M. A. *The Mammals of Russia and Adjacent Territories. Lagomorphs and Rodents* (Russian Academy of Sciences, Zoological Institute St. Petersburg, 1995).

167. Heptner, V. G. & Sludskii, A. A. *Mammals of the Soviet Union. Volume II, Part 2. Carnivora (Hyaenas and Cats)* (Smithsonian Institution Libraries and The National Science Foundation, 1992).

168. Kojamkulova, B. S. *The Antropogen Fossils of Kazakhstan* (Academy of Sciences of Kazakh SSR 1969).

169. Kozhamkulova, B. S. Pleistocene macrotheriofauna of Kazakhstan (Leading forms, artifacts). *Trudy Instituta Zoologii, Ministerstvo obrazovanya i nauki Respubliki Kazakhstan* **49**, 43-48 (2005).

170. Kozhamkulova, A. A. & Kosteňko, N. N. *Extinct Animals of Kazakhstan (Paleogeography of Late Cainozoic).* (IzdatelstvoNauka Kazachstoj SSR, 1984).

171. Vorobeev, G. G. & van der Ven, J. *Looking at Mammals in Kyrgyzia (Central Asia)* (BDC, 2003).

172. Aristov, A. A. & Baryshnikov, G. F. *The Mammals of Russia and Adjacent Territories. Carnivores and Pinnipeds* (Izdatel'stvo Zoologicheskogo Instituta RAN, 2001).

173. Bachura, O. & Kosintsev, P. Late Pleistocene and Holocene small- and large-mammal faunas from the Northern Urals. *Quat. Int.* **160**, 121-128 (2007).

174. Kosintsev, P. A. Late Pleistocene megamammals of the Urals. *Acta Zool. Cracov.* **39**, 245-250 (1996).

175. Kosintsev, P. A. Late Pleistocene large mammal faunas from the Urals. *Quat. Int.* **160**, 112-120 (2007).

176. Kuzmin, Y. V., Orlova, L. A., Stuart, A. J., Zenin, V. N. & Dementiev, N. Human-mammoth (*Mammuthus primigenius* Blumenbach) inter-relationships in the Palaeolithic of Siberia: a review of current knowledge. *The World of Elephants - International Congress, Rome 2001*,714-717 (2001).

177. Stuart, A. J., Kosintsev, P. A., Higham, T. F. G. & Lister, A. M. Pleistocene to Holocene extinction dynamics in giant deer and woolly mammoth. *Nature* **431**, 684-689 (2004).

178. Borodin, A. V., Strukova, T. V., Kosintsev, P. A., Nekrasov, A. E. & Panova, N. K. New data to characterize the Middle Urals environments of the Late Pleistocene time (the site of Shaitanoozersky Kamenny Ostrov). *Pleistocene and*

Holocene Urals Faunas (Institute of Plant and Animal Ecology, RAS, Čeljinsk) **56**, 17-35 (2000).

179. Kosintcev, P. A. in *The Holocene History of the European Vertebrate Fauna. Modern Aspects of Research* (ed. Benecke, N.) Ch. 10, 133-139 (Verlag Marie Leidorf GmbH, 1999).

180. Mosin, V. The southern Transurals in the stone age. *Izvesti Celjabinskogo naucnogo scentra* **2**, 82-83 (2000).

181. Pacher, M. *et al.* Cave bears of the Ural Mountains - a survey based on direct radiocarbon dates, aDNA and morphometrical analysis. *Abstract Book (15th International Cave Bear Symposium - Spišská Nová Ves, Slovakia, 17th - 20th of September 2009)*,14-17 (2009).

182. Alexeeva, N. V. & Erbajeva, M. A. Diversity of Late Neogene-Pleistocene small mammals of the Baikalian region and implications for paleoenviroment and biostratigraphy: An overview. *Quat. Int.* **179**, 190-195.

183. Andrenko, O. V., Ovodov, N. D., Zazhigin, V. S. & Chekha, V. P. Quaternary rodents of the NE Part of the Altai-Sayan Mountain Region. *Antropozoikum* **23**, 117-118 (1999).

184. Boeskorov, G. G. Systematics and distribution of sheep of the genus *Ovis* (Artiodactyla, Bovidae) in Eastern Siberia and the Far East in the Pleistocene and Holocene. *Zool. Zhurnal* **80**, 243-256 (2001).

185. Chlachula, J. Pleistocene climate change, natural enviroments and palaeolithic occupation of the Altai area, west-central Siberia. *Quat. Int.* **80-81**, 131-167 (2001a).

186. Chlachula, J. Pleistocene climate change, natural enviroments and paleolithic occupation of the Angara-Baikal area, east Central Siberia. *Quat. Int.* **80-81**, 69-92 (2001b).

187. Erbajeva, M., Alexeeva, N. & Khenzykhenova, F. Review of the Pliocene-Pleistocene arvicolids of the Baikalian region. *Palaeontographica A* **278**, 113-123 (2006).

188. Foronova, I. V. Quaternary mammals and stratigraphy of the Kuznetsk Basin (South-Western Siberia). *Antropozoium* **23**, 71-97 (1999).

189. Geiser, F. *Checkliste der Säugetierarten des Altai nach Belegen des Siberian Zoological Museum in Novosibirsk: Gebiete Altai Republic und Altaiskyi Krai territory* (2002) Date of access:10/02/2015

190. Erbajeva, M. A., Khenzykhenova, F. I. & Alexeeva, N. V. Late Pleistocene and Holocene environmental peculiarity of the Baikalian region, based on mammal associations and deposits. *Quat. Int.* **237**, 39-44 (2011).

191. Khenzykhenova, F. Late Pleistocene small mammals from the Baikal region (Russia). *Acta Zool. Cracov.* **39**, 229-234 (1996).

192. Khenzykhenova, F. Pleistocene disharmonious faunas of the Baikal region (Russian, Siberia) and their implication for palaeography. *Antropozoikum* **23**, 119-124 (1999).

193. Khenzykhenova, F. I. Paleoenviroments of Palaeolithic humans in the Baikal region. *Quat. Int.* **179**, 53-57 (2008).

194. Khenzykhenova, F. Mammoth and associated mammal fossils from the Lake Baikal region. *International Symposium Siberia and Japan in the Late Paleolithic period. Adaptive strategies of humans in the Last Glacial Period, Keio University*, 47-48 (2010).

195. Khenzykhenova, F. *et al.* Upper paleolithic mammal fauna of the Baikal region, east Siberia (new data). *Quat. Int.* **231**, 50-54 (2011).

196. Knapp, M. *et al.* First DNA sequences from Asian cave bear fossils reveal deep divergences and complex phylogeographic patterns. *Mol. Ecol.* **18**, 1225-1238 (2009).

197. Kuzmin, Y. V. Siberia at the Last Glacial Maximum: environment and archaeology. *J. Archaeol. Res.* **16**, 163-221 (2008).

198. Lazarev, P. A. *Large Mammals of the Yakutian Antropogene* (diss.rsl.ru, 2006).

199. Lbova, L. V. The palaeoecological model of the Upper Palaeolithic site Kamenka (Buryatia-Siberia). *Antropozoikum* **23**, 181-191 (1999).

200. Lister, A. The impact of Quaternary Ice Ages on mammalian evolution. *Philos. Trans. R. Soc. Lond. B Biol. Sci.* **359**, 221-241 (2004).

201. Motuzko, A. N., Vasilev, S. J., Vashkov, A. A., Elenskiy, J. N. & Kravchenko, E. N. The geological and geomorphological characteristic of sites of mammoth fauna of Late Pleistocene in area of foothills of East Sayan. *IV International mammoth conference*, *Yakutsk 2007*, 186-187 (2007).

202. Rzebik-Kowalska, B. New data on Soricimorpha (Lipotyphla, Mammalia) from the Pliocene and Pleistocene of Transbaikalia and Irkutsk Region (Russia). *Acta Zool. Cracov.* **50**, 15-48 (2007).

203. Rzebik-Kowalska, B. Insectivores (Soricomorpha, Mammalia) from the Pliocene and Pleistocene of Transbaikalia and Irkutsk region (Russia). *Quat Int.* **179**, 96-100 (2008).

204. Sato, T. *et al.* Vertebrate fossils excavated from the Bol´shoj Naryn site, East Siberia. *Quat. Int.* **179**, 101-107 (2008).

205. Serdyuk, N. V. Paleoreconstruction of Pleistocene environments of human habitats in the Late Pleistocene and Holocene near the Charyshskii Naves Cave, Central Altai, Russia. *Paleontol. J.* **40**, 501-507 (2006).

206. Vasiliev, S. K., Derevianko, A. P. & Markin S. V. Large mammal fauna of the Sartan period from the northwestern Altai (based on material from Kaminnaya Cave). *Archaeology, Ethnology and Anthropology of Eurasia* **2**, 2-22 (2006).

207. Vasiliev, S. K., Serdyuk, N. V. & Orlova, L. A. Late Pleistocene fauna of mammals from hyena den cave (North-west Altai). *IV International mammoth conference*, *Yakutsk 2007*, 139-140 (2007).

208. Yudin, B. S., Galkina, L. I. & Potapkina, A. F. *Mammals of the Altai-Sayan Mountainous Part* (Nauka, 1979).

209. Erbajeva, M. A. & Alexeeva, N. V. Pliocene and Pleistocene biostratigraphic succession of Transbaikalia with emphasis on small mammals. *Quat. Int.* **68-71**, 67-75 (2000).

210. Lister, A. M. The evolution of the giant deer, *Megaloceros giganteus*

(Blumenbach). *Zool. J. Linn. Soc.* **112**, 65-100 (1994).

211. Vangengeim, E. A. *Paleontologic Foundation of the Anthropogene Stratigraphy of Northern Asia (on Mammals)* (Nauka, 1977).

212. Baryshnikov, G. F. Chronological and geographical variability of *Crocuta spelaea* (Carnivora, Hyaenidae) from the Pleistocene of Russia. *Deinsea* **6**, 155-173 (1999).

213. Bocherens, H., Pacaud, G., Lazarev, P. A. & Mariotti, A. Stable isotope abundances (^{13}C, ^{15}N) in collagen and soft tissues from Pleistocene mammals from Yakutia: implications for the paleobiology of the mammoth steppe. *Palaeogeogr. Palaeoclimatol. Palaeoecol.* **126**, 31-44 (1996).

214. Boeskorov, G. G. Taxonomic Position of the Red Deer *Cervus elaphus* L. (Cervidae, Artiodactyla, Mammalia) from the Neopleistocene of Northeastern Asia. *Paleontol. J.* **39**, 73-84 (2005).

215. Boeskorov, G. G. Arctic Siberia: refuge of the Mammoth fauna in the Holocene. *Quat. Int.* **142-143**, 119-123 (2006).

216. Boeskorov, G. G. & Mol, D. Quaternary mammal collections in the Museum of Yakutsk (Eastern Siberia, Yakutia, Russia). *Cranium* **21**, 19-32 (2004).

217. Lazarev, P. A. & Tomskaja, A. I. *Mammals and Biostratigraphy of the Late Pleistocene of the Northern Yakutia* (Akademija nauk SSSR, 1987).

218. Lazarev, P. *et al. Mammals of Antropogene of Yakutia* (Yakut Scientific Centre SD RAS, 1998).

219. Nikolskiy, P. A. Late Pleistocene mammals of New Siberia Island (Russian Arctic). *32nd Annual Arctic Workshop Abstracts*, 3 (2002).

220. Zazula, G. D., Froese, D. G., Elias, S. A., Kuzmina, S. & Mathewes, R. W. Arctic ground squirrels of the mammoth-steppe. Paleoecology of Late Pleistocene middens (~24000-29 450 ^{14}C yr BP), Yukon Territory, Canada. *Quat. Sci. Rev.* **26**, 979-1003 (2007).
